# Electrical Conductivity of Multiwall Carbon Nanotube Bundles Contacting with Metal Electrodes by Nano Manipulators inside SEM

**DOI:** 10.3390/nano11051290

**Published:** 2021-05-13

**Authors:** Quan Yang, Li Ma, Shungen Xiao, Dongxing Zhang, Aristide Djoulde, Maosheng Ye, Yini Lin, Songchao Geng, Xuan Li, Tao Chen, Lining Sun

**Affiliations:** 1 School of Mechatronic Engineering and Automation, Shanghai University, Shanghai 200444, China; aidi168@shu.edu.cn (Q.Y.); djouldearistide@yahoo.fr (A.D.); yemaosheng@shu.edu.cn (M.Y.); xenia@shu.edu.cn (Y.L.); gsc9428@shu.edu.cn (S.G.); 2 School of Information, Mechanical and Electrical Engineering, Ningde Normal University, Ningde 352100, China; 3 Shenzhen Institute for Advanced Study, University of Electronics Science and Technology of China, Shenzhen 518110, China; zhangdongxing@uestc.edu.cn; 4 Robotics and Microsystems Centre, Soochow University, Suzhou 215021, China; sln@hit.edu.cn

**Keywords:** carbon nanotube, metallicity, semiconductivity, molecular dynamics, density functional theory

## Abstract

Determining the metallicity and semiconductivity of a multi-walled carbon nanotube (MWCNT) bundle plays a particularly vital role in its interconnection with the metal electrode of an integrated circuit. In this paper, an effective method is proposed to determine the electrical transport properties of an MWCNT bundle using a current–voltage characteristic curve during its electrical breakdown. We established the reliable electrical nanoscale contact between the MWCNT bundle and metal electrode using a robotic manipulation system under scanning electron microscope (SEM) vacuum conditions. The experimental results show that the current–voltage curve appears as saw-tooth-like current changes including up and down steps, which signify the conductance and breakdown of carbon shells in the MWCNT bundle, respectively. Additionally, the power law nonlinear behavior of the current–voltage curve indicates that the MWCNT bundle is semiconducting. The molecular dynamics simulation explains that the electron transport between the inner carbon shells, between the outermost carbon shells and gold metal electrode and between the outermost carbons shells of two adjacent individual three-walled carbon nanotubes (TWCNTs) is through their radial deformation. Density functional theory (DFT) calculations elucidate the electron transport mechanism between the gold surface and double-wall carbon nanotube (DWCNT) and between the inner and outermost carbon shells of DWCNT using the charge density difference, electrostatic potential and partial density of states.

## 1. Introduction

Individual semiconducting single-walled carbon nanotubes (SWCNTs) have been selected as the channel materials to build field effect transistors (FETs) and microprocessors due to their extremely high carrier mobility [1,2]. The SWCNTs can be either metallic or semiconducting according to their chirality [3], and the contact resistance between metallic SWCNTs and metal electrodes is large; there will be a large Schottky barrier at the contact interface between semiconducting SWCNTs and metal electrodes [4,5]. The metallicity and semiconductivity of multi-walled carbon nanotubes (MWCNTs) are determined by the electrical transport properties of the outermost shell and the intershell interactions [6,7,8,9]. As the scattering electrons (current/charge) are transported from the outermost carbon shell to the inner carbon shells—equivalent to increasing the conduction channels of MWCNT [3,10] making the understanding of MWCNT conduction is complex. In addition, the SWCNTs inherently tend to form an aggregated bundle as a result of van der Waals adhesion [11,12]. Therefore, it is important but challenging to obtain an individual semiconducting SWCNT from its bundle for CNTFETs fabrication [1,13]. However, SWCNTs bundles have great advantages related to the interconnection of integrated circuits. The MWCNTs also tend to aggregate together as a result of van der Waals force, forming an MWCNT bundle [8,14,15,16]. In this case, the formed MWCNT bundle structure further increases the electron conduction channels between MWCNTs and metal electrodes, thus reducing the contact resistance [17]. Therefore, the SWCNT bundles, individual MWCNTs and MWCNT bundles are proposed as possible replacements of copper wire to achieve interconnection with metal electrodes in integrated circuits [15]. However, few studies are reported on the metallicity and semiconductivity properties of MWCNT bundles contacting with metal electrodes.

The general method to determine the electrical transport properties of MWCNTs is to measure bias voltage and the current simultaneously [18,19]. If the linear current voltage curve is obtained, the contact between MWCNTs and metal electrode is ohmic, which means the MWCNTs are metallic [20]. For the clean and good contact interface, the nonlinear current voltage curve demonstrates that the MWCNTs contacting with the metal electrode are semiconducting. The methods of establishing the reliable and naturally formed electrical contact between MWCNT and metal electrodes consist of in-situ electrode growth [21], dielectrophoretic [13,18] and solution deposition [22,23]. However, once the electrical contact is established, the contact parameters and the nanodevices properties are unchangeable. A simple and effective probe directly contact method is proposed through the AFMSEM based nanoscale manipulation robotic system [24,25] to characterize the electrical conductivity of MWCNT bundles [8,16,17,19,26,27]. The voltage current characteristic curves measured at the naturally formed contact and tip directly contact are influenced by the nanoscale interface contact behaviors. However, the voltage current curve can externally and macroscopically characterizes the electrical contact behavior between MWCNTs and metal electrode. For example, some references [28,29,30,31] only gave speculative explanation for the current injection mechanism between MWCNTs and metal surface, as well as between inner shells inside MWCNT bundles. Unfortunately, it was difficult to observe the atomic interface contact behavior between MWCNTs and metal electrode effectively and experimentally [32,33,34,35] under SEM. The atomic scale molecular dynamics method has been utilized for researching the interface contact behavior of individual SWCNT [36,37], SWCNT bundle [38] and individual MWCNTs [39] with metal surface, but the research on the interfacial contact behavior of MWCNTs bundle and metal electrodes is still relatively lacking. In addition, the density functional theory (DFT) has been utilized for researching the electrical properties at the contact interface between SWCNT and metal electrode [40,41,42,43], but the electron transport mechanism between MWCNT and metal electrode or between inner carbon shells of MWCNT is rarely studied.

In this paper, a reliable electrical contact between MWCNT bundles and various metal electrodes was established via an AFMSEM-based micromanipulation robotic system, and the metallicity and semiconductivity of MWCNT bundles were determined according to the current–voltage characteristic curve corresponding to the breakdown process of the MWCNT. In addition, the electrical transport property is explained at both the atomic and electronic scale. The interface contact behavior between the TWCNT bundle and metal electrode was studied via the molecular dynamics method. The electrons’ injection mechanism between the DWCNTs bundle and metal electrode surface and between inner shells of DWCNTs was studied using DFT.

## 2. Materials and Methods

**Figure 1:**
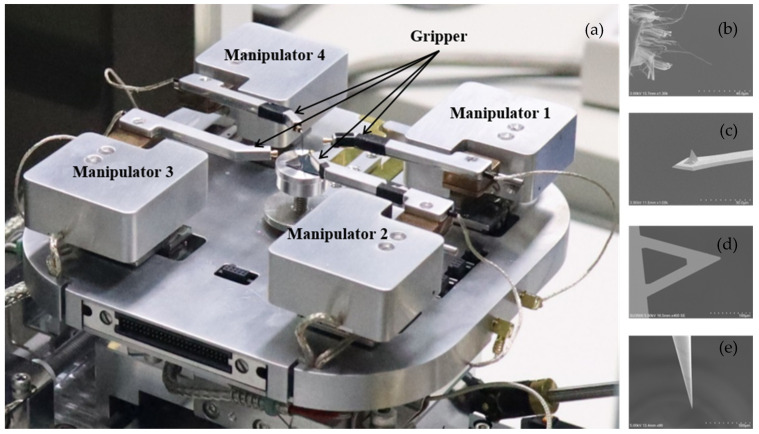
(**a**) AFMSEM-based nanoscale robotic manipulator system, (**b**) MWCNT bundle sample; (**c**) gold-coated AFM cantilever with tip; (**d**) tip-less gold-coated AFM cantilever; (**e**) tungsten probe.

To combine the real-time observational advantage of SEM and a nanoscale conductive AFM probe, an integrated AFMSEM system has previously been reported by placing several conductive AFM probes onto the gripper of each nanoscale manipulator inside an SEM chamber. Figure 1a illustrates the integrated AFMSEM-based robotic micromanipulation system, which mainly consists of micro robotic manipulation systems with four piezo-driven manipulators. To obtain real-time imaging, an SEM (SU3500, Hitachi, Tokyo, Japan) with a secondary electron detector is employed as the observation system, acquiring high-resolution images of the conductive probe. 

The individual MWCNTs originated from the vertical array forest produced by chemical vapor deposition, as shown in Figure 1b, with length ranging from 50 to 1000 µm and diameter ranging from 3 to 10 nm. The MWCNT may contain a number of walls ranging from 3 to 7. Considering the diameter parameter, each bundle may contain hundreds of individual MWCNTs. The MWCNT bundles were transferred into the SEM chamber by dipping the dispersed powder on the silicon substrate using a tungsten probe, resulting in the MWCNT bundle making contact with the tungsten probe both electrically and mechanically.

For nanoscale manipulation and electrical characterization of MWCNT bundles, the use of end effectors is the core technique for a AFMSEM-based nanoscale robotic manipulation system. As shown in Figure 1c–e, the conductive probes are a gold-coated AFM cantilever (HQ: NSC14/Au, MikroMasch, Tallinn, Estonia), gold-coated tip-less AFM cantilever (PNP-TR-TL-Au, Nanoworld, Neuchatel, Switzerland), and tungsten probe (ST-20-0.5, GGB Industries, Naples, FL, USA).

The electrical transport characteristics analysis of MWCNT bundles and metal electrodes was performed in single-electrode contact mode and double-electrode contact mode. In the single-electrode contact mode, the four kinds of end effectors, namely, a gold-plated AFM cantilever, tip-less AFM cantilever, tungsten probe and platinum-coated tungsten probe, were utilized to contact the MWCNT bundles on the substrate. After establishing a reliable mechanical and electrical contact between the MWCNT bundles and electrical conductive probes, the Keithley 2280S system source meter was used to simultaneously measure the voltage and current data. The relationship of current versus time was also obtained during the complete breakdown process of MWCNT bundles. In the double-electrode mode, MWCNT bundles were picked up from the substrate, and then moved so as to make contact with other metal electrodes, forming the electrode–bundle–electrode structure. In the same way, the current and voltage were recorded throughout the breakdown process of MWCNT bundles. The current that passed through the contact interface between the MWCNT bundle and metal electrode was measured through the BNC (Bayonet Nut Connector) coaxial port of the controller of the AFMSEM-based nanoscale manipulator robotic system. Thus, the voltage–current characteristic curves were determined to characterize the electrical contact properties between MWCNT bundles and metal electrodes.

The molecular dynamics method was utilized to explain how the atomic interface behaviors of MWCNT bundles contacting with gold electrodes influence the electron transport. Here, TWCNTs were used instead of MWCNTs to study the radial compression deformation behavior, with consideration of the computational cost. The diameter of TWCNT (10, 10) was 1.34 nm, and the layer spacing was 0.34 nm. A gold supercell with the size of 57.68 Å × 57.68 Å × 75.77 Å was constructed using the (1, 0, 0) lattice plane with a thickness of 15 Å. The key factor of a molecular dynamics calculation is the selection of the force field. Thus, all of the molecular dynamics calculations were performed using a COMPASS (Condensed-Phase Optimization Molecular Potential for Atomic Simulation Studies) force field. After determining the force field, the constant NVT (N is the number of atoms, V is the volume, T is the temperature) ensemble was chosen for the molecular dynamics simulation. In addition, the Andersen method was used to adjust the ensemble temperature, and the molecular dynamics calculation was performed at a temperature of 300 K. In order to obtain higher accuracy, the cut-off distance was 15.5 Å. To achieve atomic energy balance as soon as possible, the Velocity Verlet algorithm was selected for numerical integration of the differential equation. To obtain a stable and accurate atomic configuration and sufficient physical data, the total simulation time was set to 100 ps, the calculation step was 1 fs, and the coordinates and velocities of all atoms were stored along with the output every 1000 steps.

DFT was utilized to explain the electron transport between the metal surface and MWCNT bundle. Considering the large computational cost of the MWCNT bundle and the similar electron transport between the outermost carbon shells of two adjacent MWCNTs, DWCNTs were selected for the DFT calculations. The inner and outer carbon shells of DWCNTs were semiconducting zigzag chiral (5, 0) SWCNT with the length of 0.426 nm and diameter of 0.391 nm. The DWCNTs were unsaturated structures, which were non-hydrogenated at both two ends, containing 64 carbon atoms. The interlayer spacing was 0.2 nm. The gold surfaces were modelled by the repeated slab geometry that contains three atomic layers, and the (1, 0, 0) plane of gold was chosen to establish a 4 × 2 supercell, with 24 gold atoms. The distance between the bottom carbon atom and the gold atom was around 0.15 nm. Geometry optimizations were applied using DFT with the Perdew–Burke–Enzerhof (PBE) generalized gradient approximation (GGA), which is implemented in the CASTEP package [44,45]. In order to reduce the plane-wave basis set of the electron system, the ion and valence electron interaction was modeled using the OTFG ultra-soft pseudopotential. The plane-wave cutoff energy was taken as 400 eV. The 2 × 4 × 2 Monokhorst–Pack *k* point was used for integration of the first Brillouin zone. Geometry optimization was performed until the energy, force and displacement on the atoms were less than 10^−5^ eV, 0.03 eV/Å and 0.001 Å, respectively. Additionally, all the stress components were less than 0.05 GPA. The tolerance in the self-consistent field calculation was 10^−6^ eV/atom. The maximum number of iterations was 100, and the BFGS algorithm was used for structural optimization.

## 3. Results and Discussion

### 3.1. Establishing Electrical Contact

The gold-coated AFM cantilever was moved to contact with the freestanding MWCNT bundles, forming the single electrode structure (tungsten-powder-bundle-gold). In this mode, the MWCNT powder has a large contact area with the tungsten probe, which can establish a reliable mechanical and electrical contact. Therefore, the interface contact behavior between the gold-plated AFM cantilever and the freestanding MWCNT bundles directly influences the electrical transport properties of MWCNT bundles.

The mechanical contact between MWCNT bundles and the AFM cantilever was solid due to the van der Waals energy with a gap on the atomic-scale of 0.34 nm, but this solid mechanical contact cannot guarantee good electrical contact. For example, the small voltage cannot make the circuit conducting since no sharp cur-rent rise was observed. After applying a relatively high voltage, a sharp current increase was observed, which confirms that a high voltage is required to make the circuit conductive [10,27]. This is because the potential barrier formed between the metal electrode and MWCNT bundles is disrupted, which increases the mobility of electrons at the interface. In some cases, the circuit is not conductive under a large applied voltage. The reason for this is that a large vertical gap exists between the MWCNT bundle and the metal electrode, which can be further adjusted by changing their contact positions using the AFMSEM-based nanoscale manipulator robotic system. Upon waiting for several minutes, the graphitization of the film at the interface of MWCNT bundle and gold metal makes the circuit conductive [30] as a result of the Joule heating effect.

**Figure 2:**
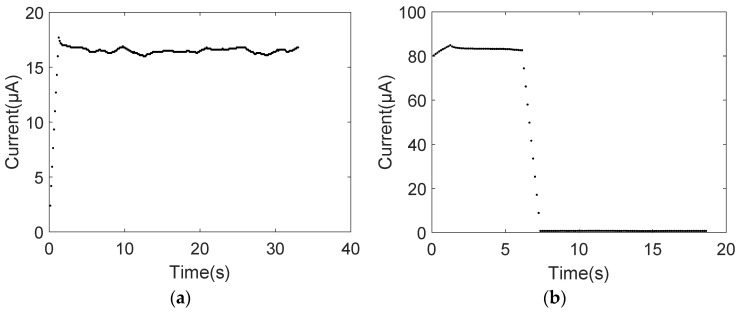
Current versus time. (**a**) The sharp current raise; (**b**) the abrupt current drop.

Figure 2a shows that the current increases sharply from zero to around 16.8 μA with a bias voltage 3 V and then remains constant. The electric circuit was conductive within about one second. However, when the bias was less than 3 V, the electrical circuit was not conductive because no obvious current change was observed. When the applied voltage increased to approximately 7.2 V, the MWCNT bundle breakdown at the middle position and the curve of current versus time is shown in Figure 2b. During the breakdown process, the abrupt current drop was from 83.4 μA to almost zero μA. However, no obvious shell-by-shell breakdown of the MWCNT bundle, as was described in [16,17,18,19], was observed here by SEM. Because the electron beam of the SEM is off in the voltage gradually increasing process. The breakdown of the whole MWCNT bundles cannot be observed neither, which can be determined through the sharp current drop.

**Figure 3:**
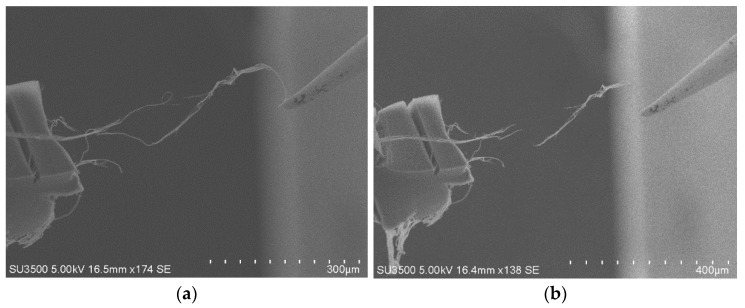
Breakdown of the bifurcated MWCNT bundle. (**a**) Breakdown of the upper part; (**b**) Breakdown of the lower part.

The SEM images in Figure 3a,b clearly show the breakdown of the bifurcated MWCNT bundle. When the applied bias was 10 V, the current was ~400 μA (25 kΩ). Then, the current dropped to ~300 μA (33 kΩ) after several seconds; at this time, the upper part of the bifurcated MWCNT bundle was broken, as shown in Figure 3a. When the bias of 12 V was provided, the current increased to ~370 μA (32.4 kΩ). When the voltage was further increased to 13 V, the current increased to ~410 μA (31.7 kΩ), and then the current dropped sharply to 0 μA. In this case, the lower part of the bifurcated MWCNT bundle also broke down, resulting in the complete breakdown of the MWCNT bundle, as shown in Figure 3b. When the upper part of the bifurcated MWCNT bundle broke down, the resistance increased. Additionally, the resistance remained constant in the same order before their second breakdown. The upper and lower parts of the bifurcated MWCNT bundle are regarded as a branch circuit and these two circuit are in parallel, thus, the voltage applied to each branch circuit was equal to the supply voltage [20].

**Figure 4:**
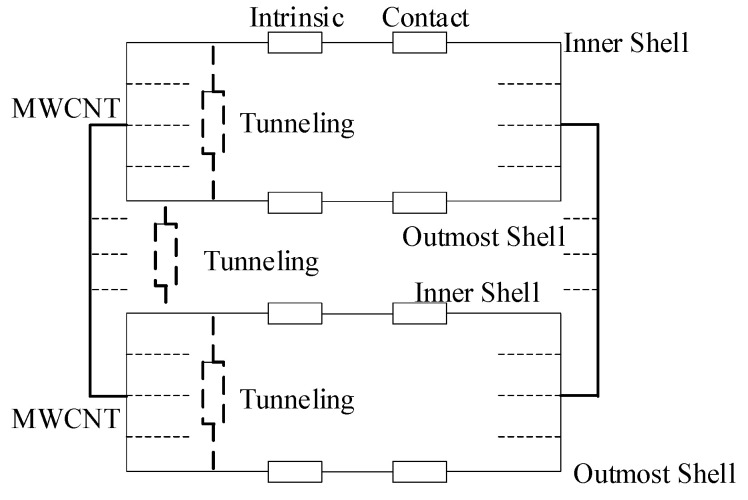
Resistance circuit model for MWCNT bundle.

Thus, according to the current change in the breakdown of the bifurcated MWCNT bundle in the Figure 3, the Figure 4 shows the resistance circuit model without considering the capacitance and inductance [15,30,46]. Additionally, each carbon shell can be regarded as a single resistance. Thus, the total resistance of an MWCNT bundle is given by the parallel combination of the resistances, including the intrinsic resistance of the MWCNT bundle, tunneling resistance between the inner carbon shells and contact resistance between the MWCNT bundle and metal electrode. Among them, the tunneling resistance mainly hinders the electron migration between inner carbon shells or between outermost carbon shells and the metal electrode. The breakdown of the MWCNT bundle means that all of the individual MWCNTs and all of the carbon shells in the individual MWCNT are conductive. However, a high applied voltage or temperature is necessary for electrons to overcome the tunneling resistance between the inner carbon shells or between the outermost carbon shells of two individual MWCNTs. Thus, the electrons can tunnel in the radial direction and then transfer along the axial direction. The electron transport mechanism in MWCNT bundles is slightly different from that in individual MWCNTs because it is more difficult for electrons to transfer between the outermost carbon shells of two adjacent individual MWCNTs than between the inner carbon shells of an individual MWCNT.

### 3.2. Single-Electrode Contact

**Figure 5:**
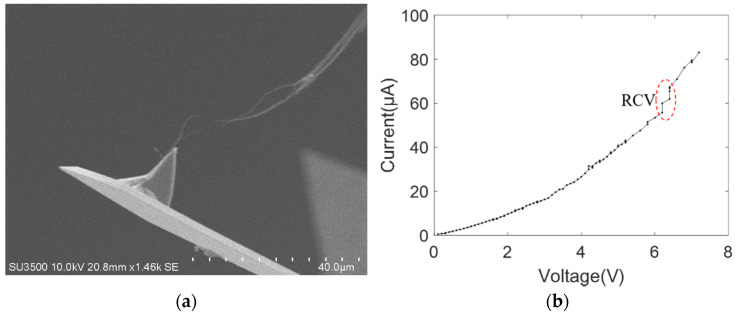
MWCNT bundle contacting with one gold-coated AFM probe (**a**) SEM image; (**b**) current–voltage curve.

Figure 5a shows the SEM image of the electrical breakdown of the MWCNT bundle contacting with the gold-coated tip of the AFM cantilever. It can be clearly observed that the MWCNT bundle breakdown occurs in the middle. We speculate that serious defects exist in this area, such as the bending of the inner carbon shell, which can narrow the current path. As the voltage increases, the current-induced Joule heating also becomes large. Thus, the high current passing through this defect area causes the MWCNT bundle to breakdown at its middle part. The breakdown behavior can reflect the local defects of the MWCNT bundle, and the current–voltage curve measured from its conductance to breakdown can determine the intrinsic electrical transport performance of the whole MWCNT bundle. Figure 5b shows the current–voltage curve measured from the conduction to breakdown of the MWCNT bundle. The nonlinear current–voltage characteristic curve indicates that the nanoscale contact between the MWCNTs and gold surface is not a simple ohmic one. However, the MWCNT bundles cannot be determined as semiconducting. This is because the powder substrate must have both metallic and semiconducting MWCNT bundles. Therefore, the current must flow from the semiconducting MWCNT to the gold-coated metal electrode. In this case, the measured current–voltage characteristic curves must be nonlinear in the single-electrode contact mode although the MWCNT bundle is metallic.

The nonlinear current–voltage curve between the MWCNT bundle and gold electrode can be described by four kinds of mathematical models, including a super linear [47] model *I* = *AV* + *BV^C^*, power law [31] model *I* = *AV^B^*, exponential law [11] model *I* = *Ae^V^*^/*B*^ and diode behavior [48,49] model *I* = *A*(*e^V^*^/*B*^ − 1), where *A*, *B* and *C* are the constants. Among them, the power law behavior of the experimentally measured current–voltage curve for the MWCNT bundle can be explained using the Luttinger liquid (LL) and environmental quantum fluctuation (EQF) theories, which can describe a one-dimensional CNT conductor. The parameter *A* is a common coefficient without a special physical meaning. The parameter *B* is the exponent of the power law, whose value is related to the contact configuration between the MWCNT bundle and metal probe, such as the probe geometry.

The power law model provided a good fit to the measured nonlinear current–voltage characteristic curve, and the fitting result was *I* = 2.3*V*^1.8^ with the goodness of fit *R*^2^ being 0.9934. This fitting result is better than that of the other three mathematical models. Additionally, the nonlinear relationship of the power law model is consistent with the fitting results of the nonlinear current–voltage characteristic curve of the MWCNT bundle given in reference [20]. Both are performed through a nanoscale manipulation robotic system under SEM to establish an electrical contact between the MWCNT bundle and the metal electrode for measuring the voltage and current. In SEM vacuum conditions, MWCNT bundles adsorb onto the metal electrode surface via the van der Waals force, which forms a weak mechanical contact with a potential barrier at the contact interface. 

**Figure 6:**
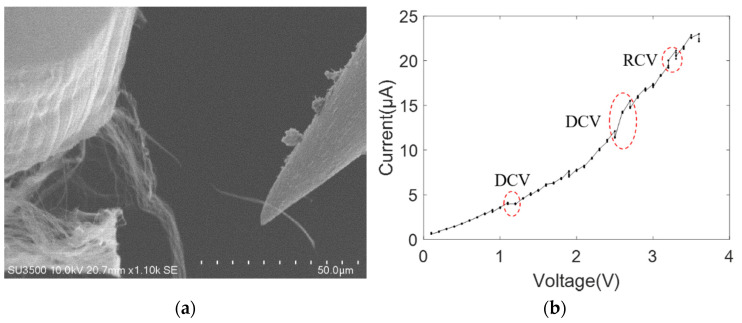
MWCNT bundle contacting with one platinum-coated tungsten probe (**a**) SEM image; (**b**) current–voltage curve.

**Figure 7:**
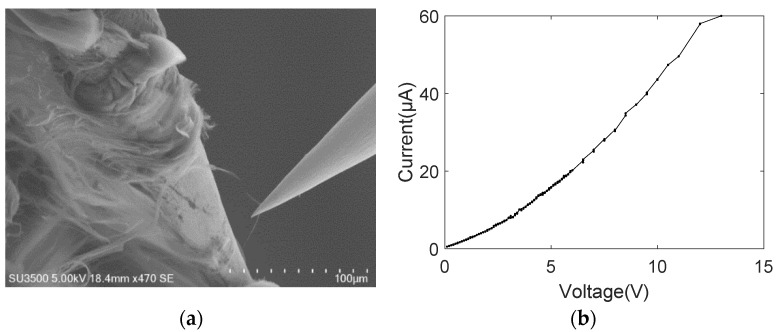
MWCNT bundle contacting with one tungsten probe (**a**) SEM image; (**b**) current–voltage curve.

The MWCNT bundles were also moved to contact with the tungsten probe and platinum-coated tungsten probe, respectively, establishing the other single-electrode contact mode. The nonlinear behavior of the current–voltage curve was observed throughout the breakdown process of MWCNT bundles, as shown in Figure 6 and Figure 7. Figure 6a shows the SEM image of the breakdown MWCNT bundle contacting with the platinum-coated tungsten probe, with the breakdown voltage and current being ~3.6 V and ~20 μA, respectively. The non-linear current–voltage characteristic curves of the platinum-coated tungsten probe and MWCNT bundles can be effectively described by the power law form, and the fitting result was *I* = 1.628*V*^1.421^ with a goodness of fit, *R*^2^, of 0.9978, as shown in Figure 6b. Similarly, Figure 7a shows the SEM image of the broken down MWCNT bundle contacting with the tungsten probe, with a breakdown voltage and current of ~12 V and ~60 μA, respectively. The nonlinear current–voltage characteristics of the tungsten probe and MWCNT bundles can also be effectively described by the power law form, and the fitting results were *I* = 2.725*V*^1.679^ with an *R*^2^ of 0.9893, as shown in Figure 7b. The exponent *B* was reported in a similar order of magnitude of about 1.8 (gold), 1.4 (platinum) and 1.7 (tungsten), which is consistent with the reported exponent in the MWCNT on the substrate [31]. This is because one end of the MWCNT bundle is on the CNT power (on-substrate) and the other end contacts with the probe (freestanding geometry) in the single-electrode contact model. Additionally, the exponent value remains almost the same in three kinds of metal materials. Thus, the parameter *B* is rarely affected by the probe materials. The AFM cantilever has a flat surface and the tungsten probe has a cone surface, thus, a large contact length and area were established at the interface of the AFM cantilever and MWCNT bundle.

### 3.3. Double-Electrode Contact

In order to avoid the influence of MWCNT powder on the conductivity of an individual MWCNT bundle, the MWCNT bundles picked up by one metal electrode (end effector, such as tungsten probe and AFM cantilever) were moved to contact with another metal electrode. In this case, the electrode–bundle–electrode structure was established, namely, the double-electrode contact model. 

**Figure 8:**
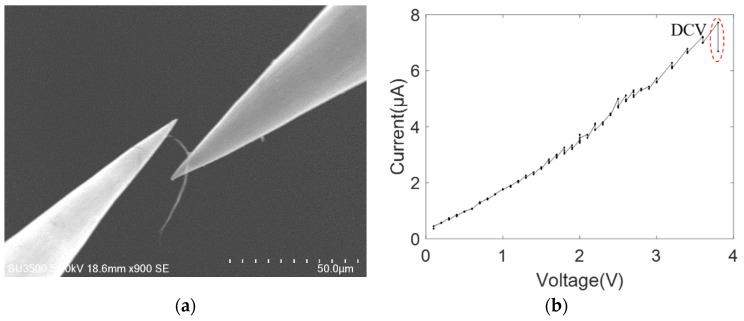
MWCNT bundle contacting with two tungsten probes (**a**) SEM image; (**b**) current–voltage curve.

As shown in Figure 8a, by moving the tungsten probe of the AFMSEM-based nanoscale manipulation robotic system, the picked-up MWCNT bundle (corresponding to the MWCNT bundle in Figure 7a) contacted with another tungsten probe. Thus, the double-electrode contact mode of tungsten–bundle–tungsten was established. As the uncertainty of MWCNT powder is avoided, the metallicity or semiconductivity of the picked-up MWCNT bundle can be determined. When the applied voltage increased to around 3.8 V, the breakdown of the MWCNT bundle at the middle position occurred, with the current being ~7.7 μA. The breakdown position was close to the left tungsten probe. Figure 8b shows the current–voltage characteristic curve of MWCNT bundles from their conduction to breakdown. The current–voltage characteristic curve was linear, and the fitting result of linear behavior was *I* = 1.932*V* − 0.145 with a goodness of fit, *R*^2^, of 0.9897. This linear behavior indicates that MWCNT bundle is metallic. The current–voltage characteristic curve of the same MWCNT bundle was nonlinear in the single-electrode contact mode but linear in the double-electrode contact mode, indicating that both metallic and semiconducting carbon nanotubes exist in the MWCNT powder.

**Figure 9:**
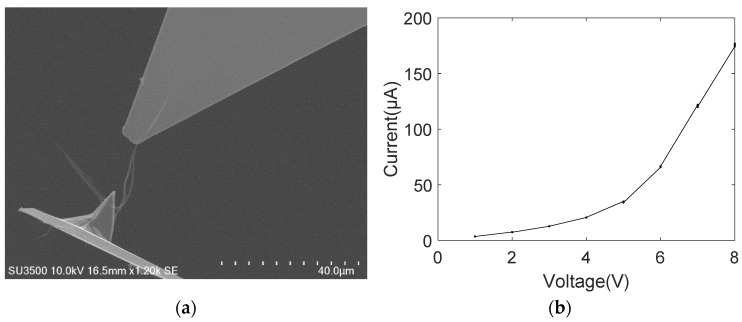
MWCNT bundle contacting with two gold-coated AFM probes (**a**) SEM image; (**b**) current–voltage curve.

Figure 9a shows the double-electrode contact mode of the gold–bundle–gold structure with the gold-coated AFM cantilever as the electrodes. The left AFM cantilever has a gold-coated tip, and the right AFM cantilever is the tip-less gold-coated cantilever. The current–voltage characteristics of this double-electrode contact structure are shown in Figure 9b. In this case, the breakdown position was near the AFM cantilever, and the breakdown voltage was approximately 8 V with the current being ~180 μA. The reason for the high current is the good electrical contact condition that was established because of the large contact area of the MWCNT bundle on the AFM cantilever surface. By contrast, the contact area of the MWCNT bundle on the tungsten probe is relatively small as a result of its cone structure. The nonlinear current–voltage characteristic is well described by the power-law behavior, with the fitting result of nonlinear behavior being *I* = 0.06*V*^2.53^ and goodness of fit, *R*^2^, of 0.9712. 

**Figure 10:**
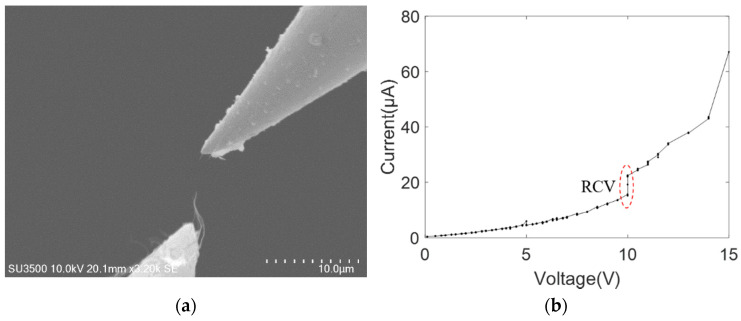
MWCNT bundle contacting with two platinum-coated tungsten probes (**a**) SEM image; (**b**) current–voltage curve.

Similarly, another double-electrode mode leading to a platinum–bundle–platinum structure was established, with two platinum-coated tungsten probes as the electrodes. Figure 10a displays the SEM image of a broken-down MWCNT bundle contacting with two platinum-coated tungsten probes. The breakdown voltage was around 15 V, with the current being ~66 μA. As shown in Figure 10b, the nonlinear current–voltage characteristic curve was also obtained in the power-law form and the fitting result was *I* = 0.229*V*^3.198^ with a goodness of fit, *R*^2^, of 0.9958.

We conclude that the MWCNT bundle can be metallic or semiconducting. The linear current–voltage characteristic curve indicates the MWCNT bundle is metallic. The semiconducting MWCNT bundles have a nonlinear current–voltage characteristic curve. In the double-electrode contact model, the exponent *B* was reported to be approximately 2.53 (gold–gold) and 3.189 (platinum–platinum). The exponents are relatively large compared with those in the single-electrode contacting model, which is consistent with the reported exponent in the freestanding MWCNT contacting with the tungsten probe under SEM vacuum conditions. In some cases, the configuration of the interface contact between MWCNT bundles and metal electrodes may affect the nonlinear behavior. Therefore, it is necessary to establish a good electrical contact configuration, involving the avoidance of the influence of impurities, oxygenates and Joule heating at the interface. 

As shown in the nonlinear current–voltage characteristic curves between MWCNT bundles and metal electrodes from Figure 5, Figure 6, Figure 7, Figure 8, Figure 9 and Figure 10, the obvious saw-tooth-like current change appears, that is, the up and down steps of current. The current up-steps are divided into the rise caused by the rising voltage (RRV) and the rise under the constant voltage (RCV). The RCV and RRV indicate whether more carbon shells or more adjacent individual MWCNTs in the bundle structure contribute to the total current. Similarly, the current down steps can be divided into the drop caused by the rising voltage (DRV) and the drop under the constant voltage (DCV). The DRV and DCV signify loss of carbon shells of MWCNT or the individual MWCNTs. However, the current–voltage characteristic curve in Figure 7b has no obvious saw-tooth-like features. This is because the applied voltage increases gradually with a small increment and a long stay time. In this case, the resulting Joule heating of the form *Q* = *UIt* changes the interface contact configuration and the internal structure of the carbon nanotubes. Additionally, no obvious saw-tooth-like features appear in Figure 9b because the height of the current up and down steps (~2 μA) cannot be clearly seen in the large current longitudinal coordinates. 

We also provide information about the other six groups of current–voltage curves (Figures S1–S6) in the single-electrode contact mode and the other three groups of current–voltage curves (Figures S7–S9) in the double-electrode contact mode in the Supplementary Materials. As shown in the 14 groups of the current–voltage curve, no obvious current up and down changes can be seen at a low voltage (1 V). This is because several individual deformed MWCNTs near the metal surface play a role in the conductance of the MWCNT bundle. As the voltage increases, there is a current change in the measured current–voltage curve, including RCV, RRV, DCV and DRV. However, the current increase is not on the same order of magnitude in the different contact condition, as shown in Table 1. In one case, the large current increases were approximately 7.4, 12, 23, 74, 124, 15 and 25.2 μA, which is due to the conductance of the amorphous carbon layer at the contact. In their research, the abrupt current increase in the current–voltage curve of MWCNTs was ~10 μA [8]. In another case, the small current increase was from 0.8 to 5 μA, which is due to the conductance of more adjacent individual MWCNTs in its bundle structure. In order to avoid experiment measurement error, the uncertainty was approximately 2.2 ± 1.5 μA. This current change is consistent with the results of ~0.8 μA in reference [49], which indicate that many shells contributed to the conductance of the MWCNT. Similarly, the current decrease, including DCV and DRV, was approximately 9.3 ± 3 μA, which means loss of carbon shells of MWCNT or the individual MWCNT. According to the parallel resistance circuit model for the MWCNT bundle, the current drop (raise) in the current–voltage curve is mainly due to the breakdown (conductance) of MWCNTs.

### 3.4. Molecular Dynamics Simulation

**Figure 11:**
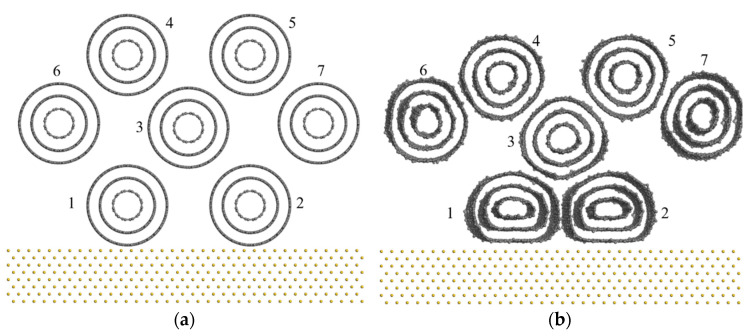
Molecular dynamics simulation of the contact behavior between MWCNT bundles and gold slab: (**a**) initial MWCNT bundle; (**b**) radially deformed MWCNT bundle.

Figure 11a shows the molecular dynamics model of MWCNT bundles contacting with the gold surface because a nonlinear current–voltage curve is rarely affected by the metal materials. The MWCNT bundle consists of seven TWCNTs with random positions. Two of them are close to the gold surface, and the other five TWCNTs are further away from the gold surface. As shown in Figure 11b, the two MWCNTs (MWCNT No.1 and MWCNT No.2) near the gold electrode surface have large radial compression deformation. The outermost carbon shell has a large direct contact area with the gold surface, and the contact distance is about 0.34 nm. This atomic-scale spacing enables electrons to transfer from the metal surface to the outermost carbon shell of these two radially deformed MWCNTs. Due to the concentric cylindrical structure of the TWCNTs (wall spacing 0.34 nm) and their deformation, the contact area between the inner carbon shells is much larger than that between the outermost carbon shell and the gold surface, which makes the electron transfer ability between inner carbon shells equal to that between metal and the outermost carbon shells. Thus, at the low voltage, these two deformed MWCNTs play the vital role in the conductance of the MWCNT bundle, without an obvious saw-tooth-like current change being observed. In reference [48], they hypothesized that the up step is because of the conductance of more inner carbon shells, but they did not consider the MWCNT deformation. For MWCNTs that are located far away from the metal surface, they only have a small degree of deformation. Thus, the contact area of their outermost carbon shells is relatively small compared with the inner carbon shells. For example, the outermost carbon shell of MWCNT No.3 has a small contact area with the four adjacent individual MWCNTs. However, its inner carbon shells almost remain parallel with the outermost carbon shells in the contact area. Therefore, the electron transport between the outermost carbons shells of two adjacent individual MWCNTs is more difficult than that between the inner carbon shells. Once the conductance of the outermost carbon shells is completed, the conductance of the inner carbon shells become easier. Therefore, the sharp current increase (RRV and RCV) in the voltage–current characteristic curve may be the result of current conductance between the outermost carbon shells of the adjacent MWCNTs with only a small degree of deformation. 

### 3.5. DFT Simulation

**Figure 12:**
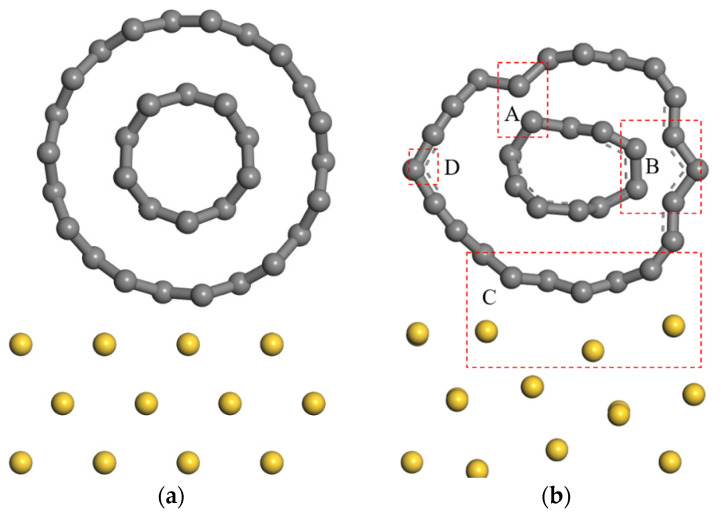
Interface contact configuration of DWCNT and gold slab (**a**) before optimization; (**b**) after optimization.

Figure 12b shows the interface contact configuration between the DWCNT and the gold surface after structural optimization. The cylindrical DWCNT experiences radial compression deformations, forming interactive carbon atom pairs between the inner and outer carbon shells. Compared with the structural optimization model of SWCNTs and gold slabs [40,42], the interaction exists between the inner and outer carbon shell as well as t between the outermost carbon shells and a gold slab in the structural optimization model of DWCNT and gold surfaces, as shown in the red rectangular box in Figure 12b. The carbon–gold distance is 0.21 nm, among which one gold atom in the first layer of the gold slab moves gradually away from the carbon atom to maintain this considerable distance. The carbon–gold distance is 0.143 nm, which is less than 0.2 nm in the initial state. The interaction distance between carbon and gold atoms is less than that of the van der Waals interaction ranging from 0.3 to 0.4 nm.

**Figure 13:**
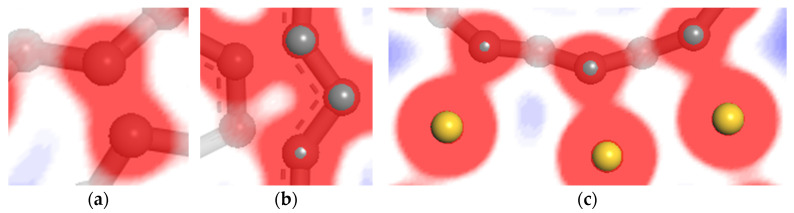
Electrostatic potential at the interface of DWCNT contacting with gold. (**a**) region A; (**b**) region B; (**c**) region C.

Figure 13 shows the 2D slice of the electrostatic potential obtained from the self-consistent field (SCF) calculation. It can be seen from Figure 13a,b that the SCF electrostatic potential of carbon atoms in the inner and outer shells of DWCNT are almost equal, and no carbon to carbon electrostatic energy barrier forms. In Figure 13b, the lower carbon atoms in the inner carbon shell and the third carbon atoms in the outer carbon shell are a pair of interacting atoms, but their SCF electrostatic potential is not equal. This is because they are not in the same plane due to the structural deformation. Figure 13c displays the SCF electrostatic potential of carbon atoms in outer shells of DWCNTs and the first layer of the gold slab. It can be seen that the electrostatic potential between carbon and gold atoms is basically the same, and no carbon to gold electrostatic energy barrier forms. Therefore, the electrons can easily migrate from the gold surface to the outer shells of DWCNTs, but this is relatively difficult compared with the electron transport between carbon atoms in the inner and outermost shells of DWCNT.

**Figure 14:**
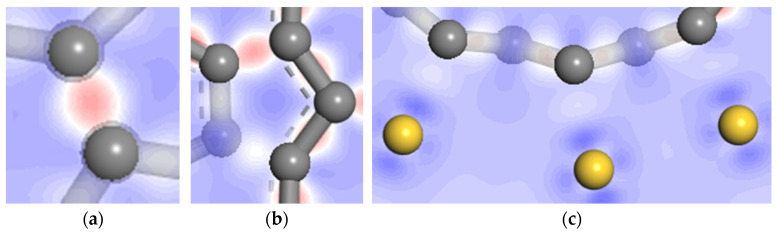
Charge density difference at the interface of DWCNT contacting with gold. (**a**) region A; (**b**) region B; (**c**) region C.

The charge density difference between carbon and gold atoms can contribute to an understanding of the electron transport mechanism at the interface between MWCNTs and metal electrodes. The charge density difference can be computed as follows: the charge density of the whole system minus the sum of the charge densities of the metal surface and SWCNTs calculated as an isolated system in the geometry position of the optimized whole system. From a slice of the charge density difference, a positive differential charge density indicates electron enrichment. Conversely, a positive differential charge density shows where the density has been depleted. Figure 14 displays a 2D slice showing the charge density difference cutting through the DWCNTs and the gold surface. It can be seen from Figure 14a,b that the charge density difference between carbon atoms is positive, indicating that a strong covalent bond is formed. The electrons gather between carbon atoms, thus, electrons are relatively easy to transfer from outer to inner carbon shells of DWCNTs. As can be seen from Figure 14c, the electron enrichment near the carbon atom and the electron dissipation near the gold atom indicate that the electrons migrate from the gold atom to the carbon atom; however, the electron migration is relatively difficult.

**Figure 15:**
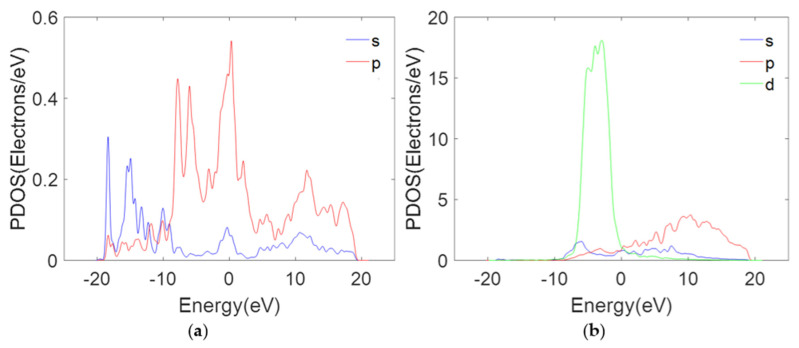
The PDOS for (**a**) the first layer of gold surface, (**b**) the carbon atoms nearest to the gold surface. Fermi energy is set to 0 eV.

The adsorption and electron transfer of DWCNTs on metal surfaces are clarified from the perspective of electron orbital hybridization. Figure 15 shows the PDOS for the first layer of a gold slab and the carbon atoms closest to the gold surface (the carbon atoms in the middle of the region C). The PDOS related to carbon atoms shows a large peak near the Fermi energy in the p-orbital, as shown in Figure 15a. As shown in Figure 15b, the PDOS of the d-orbitals of the first layer of the gold surface is large compared to that of p- and s-orbital orbits on the Fermi surface. Therefore, the p-orbital of carbon atoms can overlap with the d-orbital of the first layer of gold atoms at the Fermi energy to realize the electron transport in a hybrid orbital [42]. In this case, the electrons can easily migrate between DWCNTs and the gold surface.

**Figure 16:**
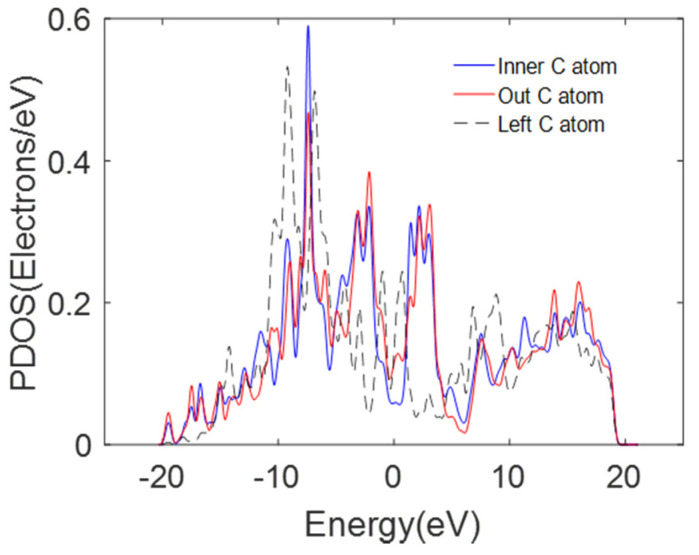
The PDOS for the carbon atoms in the DWCNT. Fermi energy is set to 0 eV.

Figure 16 shows the PDOS of p-orbitals associated with carbon atoms in the inner and outermost carbon shells of DWCNTs in which covalent bonds are formed. As shown in Figure 16, the p-orbits of two inner and outermost carbon atoms in region A have an almost identical energy distribution interval and PDOS [50]. Thus, the inner carbon p-orbitals are available to overlap with the outer carbon p-orbitals to share electrons. However, the p-orbitals of the outermost left carbon atom cannot overlap in region D (shown in Figure 12b) as it is far away from these two carbon atoms. Therefore, the strong interaction between the two carbon atoms hybridizes their p-orbitals. Orbital hybridization is favorable for electron transport between the inner and outermost carbon shells. The electron transfer may follow the above-mentioned principle between the outermost carbon shells of individual MWCNTs in the bundle structure.

## 4. Conclusions

The electrical conductivity of MWCNT bundles contacting with several kinds of metal was determined using an AFMSEM-based nanoscale manipulation robotic system. The current–voltage curve of MWCNT bundles in single-electrode contact mode (tungsten, platinum and gold) is always the nonlinear as a result of contacting with MWCNT powder. The current–voltage curve of MWCNT bundles in double-electrode contact mode is either linear or nonlinear, which can illustrate that the picked-up MWCNT bundles can be metallic or semiconducting. Through analysis of the saw-tooth-like current changes including up and down steps, the electrical conduction and breakdown of MWCNT bundles was determined. The molecular dynamic simulation shows that electron transport between the inner carbon shells is easier than that between the outermost carbon shells and gold metal electrode and between the outermost carbons shells of two adjacent individual MWCNTs. The DFT calculations show that the electrons can easily migrate from the gold surface to the outermost shells of DWCNTs, but this is relatively difficult compared with electron transport between the inner and outermost carbon shells of DWCNTs.

## Figures and Tables

**Table 1:** The current change value in current–voltage curve.

Group	RCV (μA)	RRV (μA)	DCV (μA)	DRV (μA)
1	23/124	/	/	/
2	4	15	/	/
3	1.1/3.2/0.8/1.8/1	/	/	/
4	12	/	/	6/13
5	/	/	8	/
6	74	/	5.6	/
7	/	25.2	13.7	/
8	/	0.2	/	/
9	1.1/0.9/1/4.7	/	/	/
Figure 5	5/5	/	/	/
Figure 6	1	1/0.7/0.8	/	/
Figure 8	/	/	1	/
Figure 9	1.2/1/2	/	/	/
Figure 10	1.5/7.4	/	/	/

## Data Availability

The data presented in this study are available on request from the corresponding author.

## Supplementary Materials

The following are available online at https://www.mdpi.com/article/10.3390/nano11051290/s1, Figure S1. AFM cantilever contacting with MWCNT bundle and the current voltage curve; Figure S2. Tip-less AFM cantilever contacting with MWCNT bundle and the current voltage curve; Figure S3. AFM cantilever contacting with MWCNT bundle and the current voltage curve; Figure S4. Tungsten probe contacting with MWCNT bundle and the current voltage curve; Figure S5. Pt-coated Tungsten probe contacting with MWCNT bundle and the current voltage curve; Figure S6. Pt-coated tungsten probe contacting with MWCNT bundle and the current voltage curve; Figure S7. Pt-coated tungsten probe-MWCNT bundle-AFM cantilever and the current voltage curve; Figure S8. Pt-coated tungsten probe-MWCNT bundle-tungsten probe and the current voltage curve; Figure S9. Tungsten probe- MWCNT bundle- AFM cantilever and the current voltage curve.
