# Supplementary material for: Electrical Conductivity of Multiwall Carbon Nanotube Bundles Contacting with Metal Electrodes by Nano Manipulators inside SEM
Source: Nanomaterials (Basel). 2021 May 13;11(5):1290. doi: 10.3390/nano11051290 (PMC8153315; doi:10.3390/nano11051290)
Supplement: Supplementary file 1 [file nanomaterials-11-01290-s001.zip › nanomaterials-1205310-supplementary.pdf]

Supplementary Materials

Electrical conductivity of multiwall carbon nanotube bundle contacting with metal electrodes by nano manipulators inside SEM

Quan Yang [1], Li Ma [1,*], Shungen Xiao [2,*], Dongxing Zhang [3], Aristide Djoulde [1], Maosheng Ye [1], Yini Lin [1], Songchao Geng [1], Xuan Li [4,*], Tao Chen [4] and Lining Sun [4]

1 School of Mechatronic Engineering and Automation, Shanghai University, Shanghai, 200444, China; aidi168@shu.edu.cn (Q.Y.)

2 School of information, Mechanical and Electrical Engineering, Ningde Normal University, Ningde, 352100, China; xiaoshungen022@163.com (S.G.X.)

3 Shenzhen Institute for Advanced Study, University of Electronics Science and Technology of China, Shenzhen, 518110, China; zhangdongxing@uestc.edu.cn (D.X.Z.)

4 Robotics and Microsystems Centre, Soochow University, Suzhou, 215021, China; sln@hit.edu.cn (L.N.S.)

* Correspondence: malian@shu.edu.cn (L.M.); xiaoshungen022@163.com (S.G.X.); xuanli@suda.edu.cn (X.L.)

We obtained about the other 6 groups of current voltage curves in the single electrode contact mode and the other 3 groups of current voltage curves in the double electrode contact mode in the Supporting Materials.

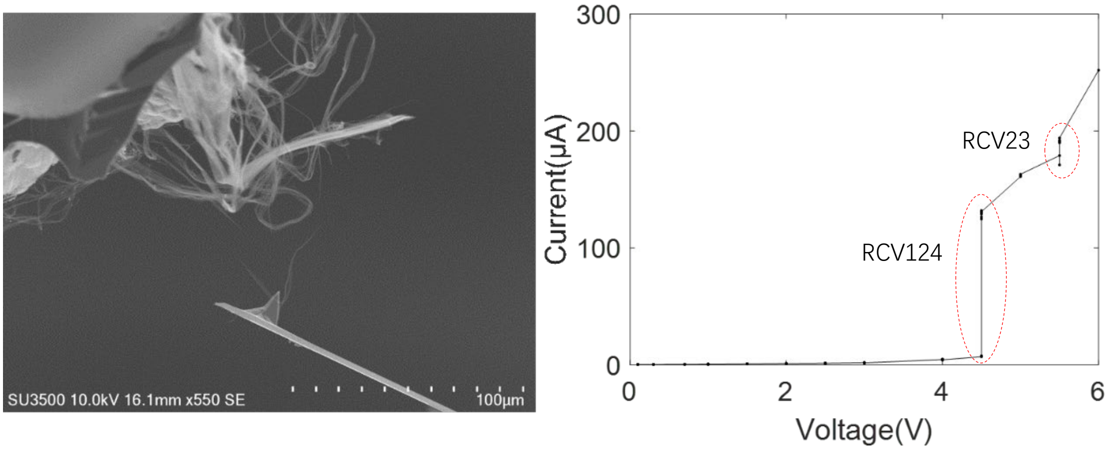

Figure S1 AFM cantilever contacting with MWCNT bundle and the current voltage curve

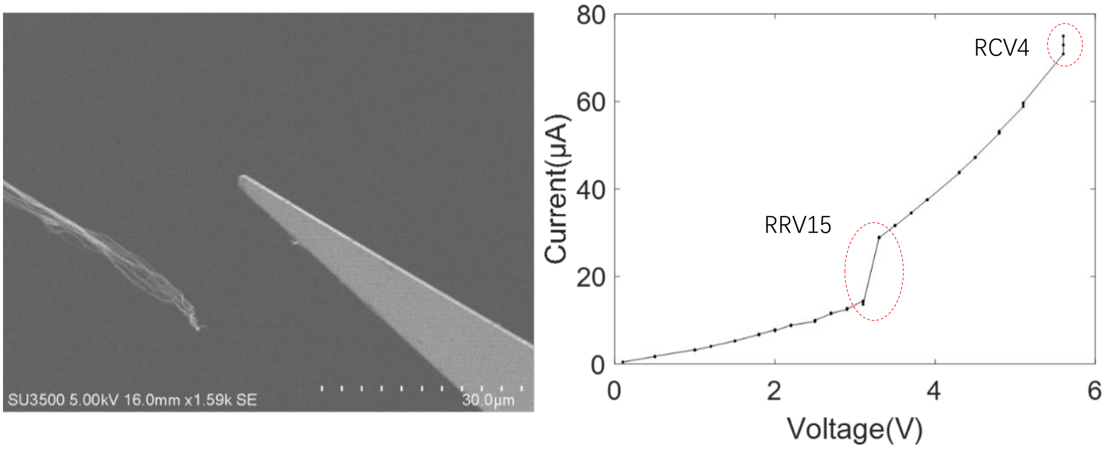

Figure S2 Tip-less AFM cantilever contacting with MWCNT bundle and the current voltage curve

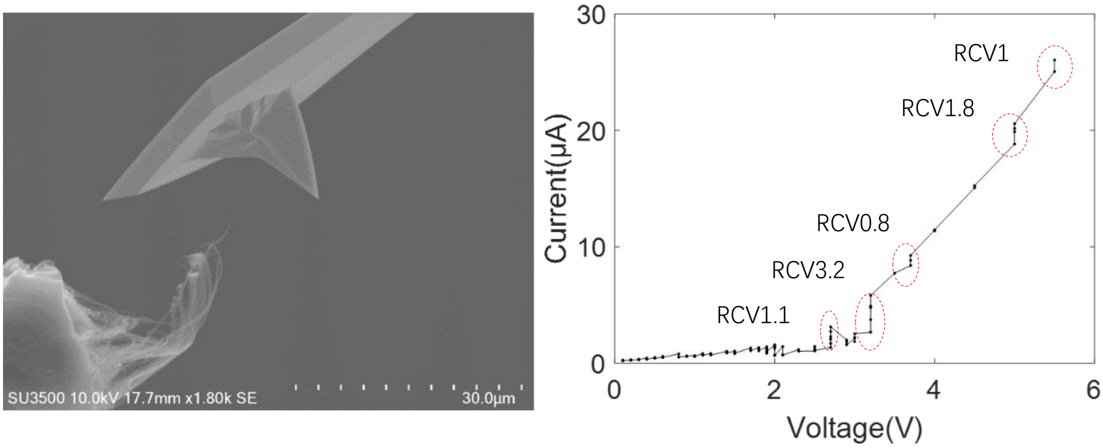

Figure S3 AFM cantilever contacting with MWCNT bundle and the current voltage curve

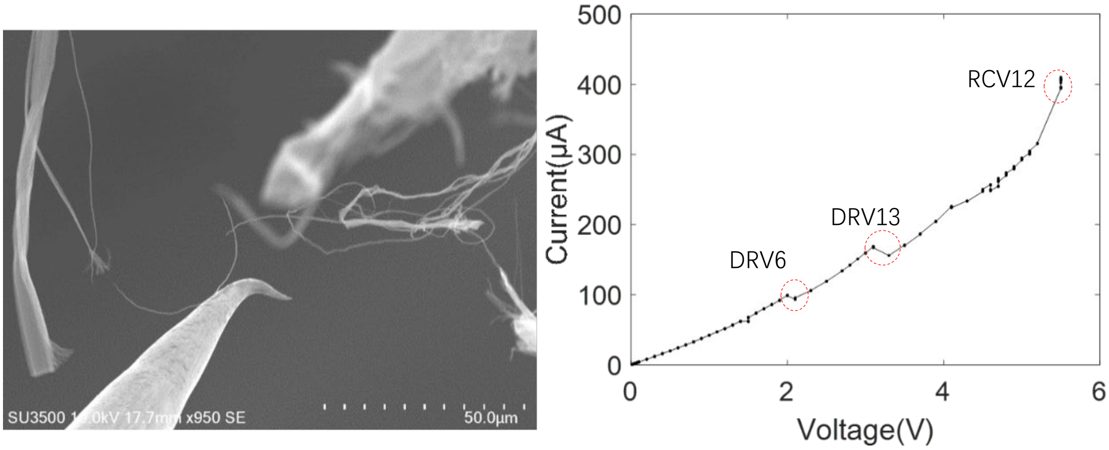

Figure S4 Tungsten probe contacting with MWCNT bundle and the current voltage curve

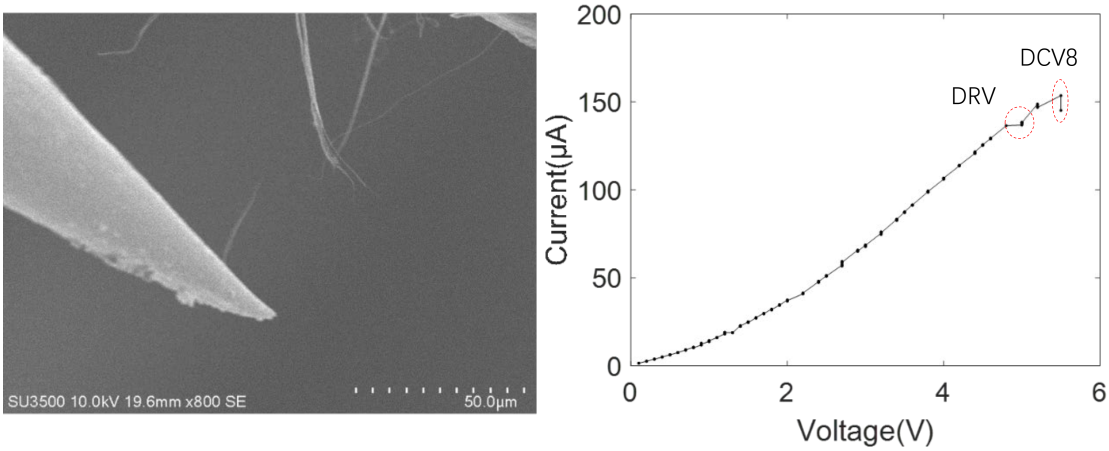

Figure S5 Pt-coated Tungsten probe contacting with MWCNT bundle and the current voltage curve

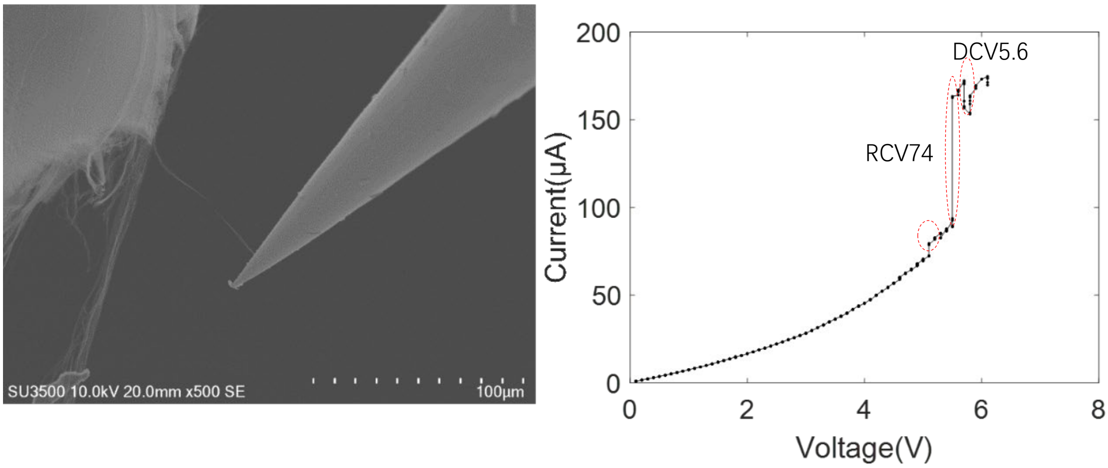

Figure S6. Pt-coated tungsten probe contacting with MWCNT bundle and the current voltage curve.

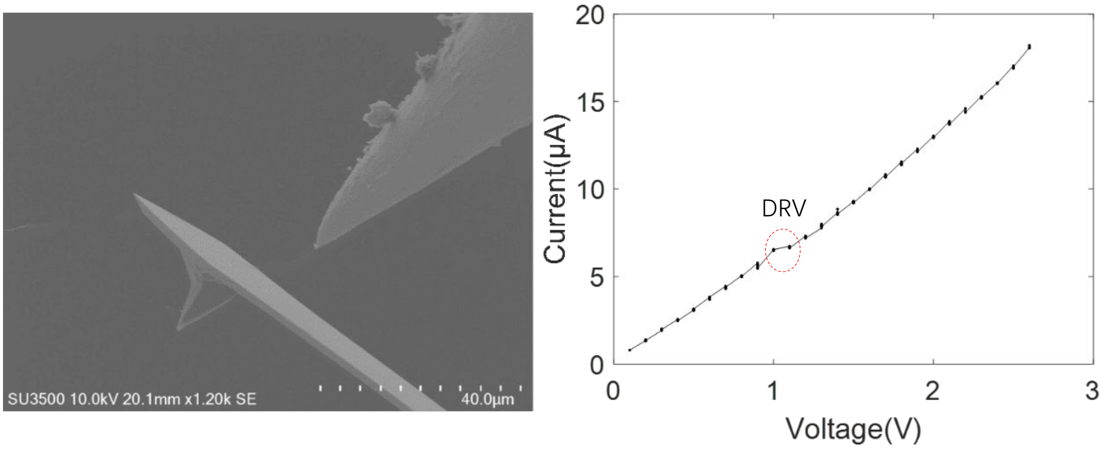

Figure S7. Pt-coated tungsten probe-MWCNT bundle-AFM cantilever and the current voltage curve.

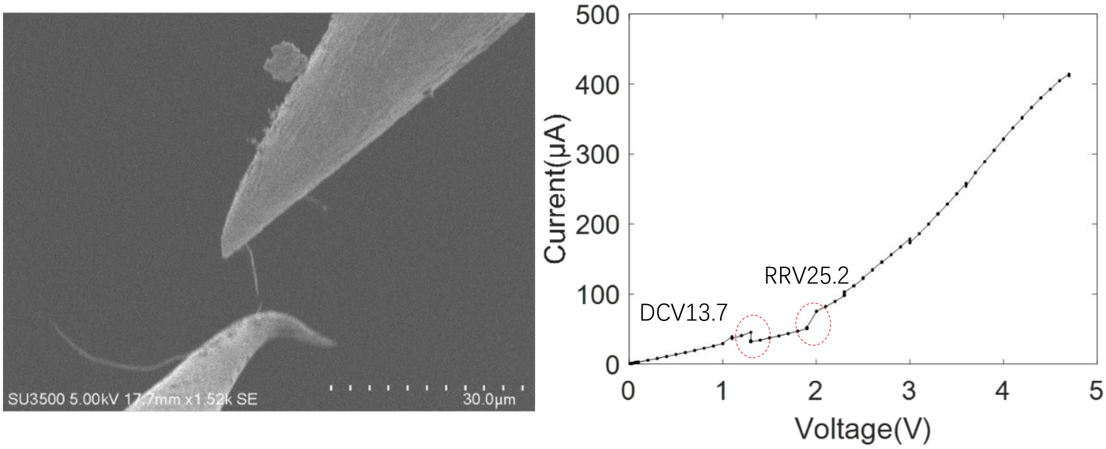

Figure S8. Pt-coated tungsten probe-MWCNT bundle-tungsten probe and the current voltage curve.

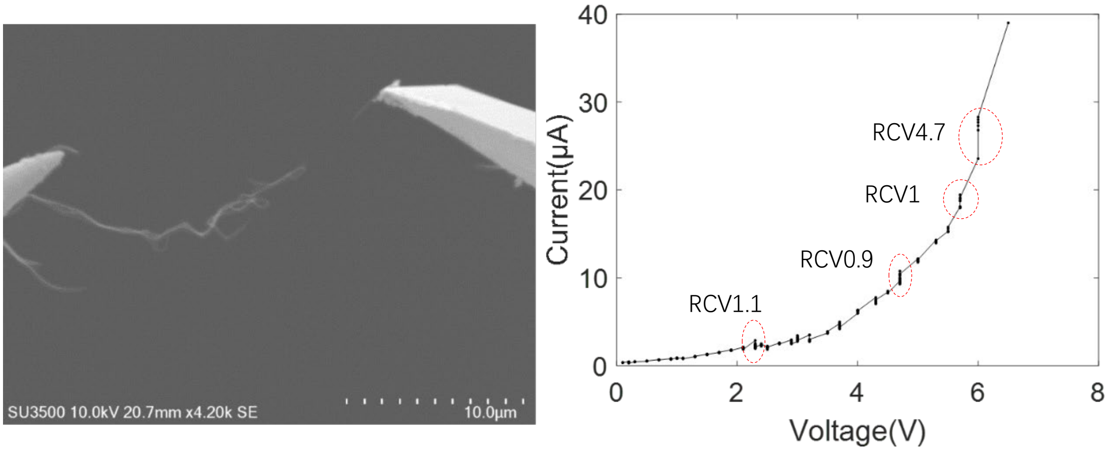

Figure S9. Tungsten probe- MWCNT bundle- AFM cantilever and the current voltage curve.
